# Information Management of Genome Enabled Data Streams for *Pseudomonas syringae* on the Pseudomonas-Plant Interaction (PPI) Website

**DOI:** 10.3390/genes2040841

**Published:** 2011-11-02

**Authors:** Magdalen Lindeberg

**Affiliations:** Department of Plant Pathology and Plant Microbe Biology, 302 Plant Science Building, Cornell University, Ithaca NY 14853, USA; E-Mail: ML16@cornell.edu; Tel.: +1-607-254-7297; Fax: +1-607-255-4471

**Keywords:** *Pseudomonas syringae*, genome annotation, PPI, type III effector

## Abstract

Genome enabled research has led to a large and ever-growing body of data on *Pseudomonas syringae* genome variation and characteristics, though systematic capture of this information to maximize access by the research community remains a significant challenge. Major *P. syringae* data streams include genome sequence data, newly identified type III effectors, biological characterization data for type III effectors, and regulatory feature characterization. To maximize data access, the Pseudomonas-Plant Interaction (PPI) website [1] is primarily focused on categorization of type III effectors and curation of effector functional data represented in the Hop database and Pseudomonas-Plant Interaction Resource, respectively. The PPI website further serves as a conduit for incorporation of new genome characterization data into the annotation records at NCBI and other data repositories, and clearinghouse for additional data sets and updates in response to the evolving needs of the research community.

## 1. Introduction

The bacterial phytopathogen, *Pseudomonas syringae*, is composed of a large number of strains having diverse and host-specific interactions with host plants and as such, has long been recognized as a valuable model system for dissecting the molecular interactions governing pathogenesis [2]. Complete genome sequences for three diverse *P. syringae* strains [3-5] together with the more recent wave of draft sequences [6-11] has provided new opportunities for characterization of *P. syringae*-host interactions; however, the systematic extraction and communication of meaningful information from the large volume of data being generated remains a significant hurdle. Indeed, *P. syringae* exemplifies the promises and challenges of using genome sequence data and other high throughput analyses to address complex molecular dynamics in host-pathogen interactions.

The *P. syringae* pan-genome has been shaped by a long-standing evolutionary arms race between the various strains and their plant hosts. Bacterial features playing a prominent role in determining the outcome of the host-pathogen interaction include conserved features of the bacterium that function as triggers for innate immunity in the host, effector proteins secreted by the type III secretion system and interfering with detection of the bacterium by the plant, toxins modulating virulence through a variety of mechanisms, and various metabolic capabilities implicated in the exploitation of the available nutrients in different plant hosts [2,12,13]. Genome sequencing has revealed the extent of strain-to-strain differences in these features, particularly among the Type III effector and toxin repertoires. Surprising levels of variation are evident even among closely related strains [8,11], generally defying a simple explanation for differences in host specificity.

Fundamental outstanding questions include some of the following. What is the repertoire of type III effectors and other factors collectively required for pathogenesis on different hosts? What is the inventory of host genes interacting with effectors and involved in defense? How are *P. syringae* isolates evolving and outcompeting one another in response to inter-strain competition and selection pressure from host plants carrying specific resistance gene profiles? Ultimately, what elements of host defense could potentially be manipulated to create more durable resistance in economically important plants? Genome-wide analyses coupled with targeted characterization of individual system components of greatest interest offer the best hope for comprehensively addressing these questions. However, the scale of data currently being generated by the “next generation” sequencing technologies in particular, presents a real challenge in how best to extract and disseminate the information contained within these datasets. This review will address some of the major types of data being generated by the *P. syringae* research community as well as strategies for maximizing accessibility to the user community.

## 2. *P. syringae* Data Streams

Data being generated by the *P. syringae* research community falls into several categories, each presenting its own particular challenges. Specific approaches in use to process these data at the Pseudomonas-Plant Interaction (PPI) website will be addressed in Section 3.

### 2.1. Genome Sequences

Genome sequencing of *P. syringae* strains continues to provide an important source of new data on the potential bases of strain to strain variation in the interactions with host plants. Complete sequences were published for *P. syringae* pv. *tomato* DC3000 (*Pto* DC3000), *P. syringae* pv. *syringae* B728a [4], and *P. syringae* pv. *phaseolicola* 1448A [5] between 2003 and 2005, with *Pto* DC3000 receiving the most comprehensive experimental characterization. Since then, advances in sequencing technology have made possible the rapid generation of draft genomes of which 13 have been released of the over 30 *P. syringae* genome projects currently registered at NCBI. These sequences represent a wealth of critical raw material for a wide range of analyses including identification of predicted virulence factors and, through genome comparison, both identification of genes linked to particular phenotypes and characterization of the evolving *P. syringae* pan-genome [14].

### 2.2. Type III Effectors—Identification in New Genomes

Of the *P. syringae* genetic components, type III effectors have been subject to the most intensive characterization owing to their central role in pathogenicity and host range. Indeed, one of the chief motivations for sequencing *Pto* DC3000 and subsequent strains has been the comprehensive identification of type III effector repertoires. Sequence features associated with the type III effector or *hop* genes (hrp outer proteins) include a binding site for the HrpL regulator upstream of the gene start, and conserved residues in the N-terminal regions of predicted Hop proteins associated with translocation by the type III secretion system. Methods for effector-mining, exploiting these features and developed using the closed genomes, have been applied to subsequent sequences [8,9].

Hop gene identification in the newly sequenced genomes adds to the expanding picture of effector repertoires in several respects, the most significant being identification of novel type III effector gene families. A recent study characterizing the effector repertoires of 14 newly sequenced *P. syringae* genomes revealed the existence of nine new *hop* gene families [11], raising the tantalizing possibility that the full range of effector-host interactions has yet to be identified. The vast majority of type III effectors encoded by the newly sequenced genomes are similar to ones previously identified, though as demonstrated for the HopZ and AvrPto families, relatively small changes in sequence can dramatically alter interactions with plant hosts [15-17]. Indeed, phenotypic differences observed for these naturally occurring variants have contributed significantly to our understanding of effector actions. Finally, genome sequencing has led to identification of large numbers of *hop* gene fragments and pseudogenes. Though not encoding functional proteins, these gene fragments provide important clues as to how effector repertoires have changed over time due to selection pressure from the host [14].

### 2.3. Type III Effectors—Biological Characterization

Identification of new type III effector families is paralleled by experimental characterization of their interactions with host plants. The generally accepted model of plant defense involves recognition of conserved elements of the bacterial cell known as pathogen associated molecular patterns or PAMPS by pattern recognition receptors, resulting in signal transduction and ultimately induction of various defense processes [18]. This process, also known as PAMP-triggered immunity or PTI, can be disrupted or suppressed at various stages by the Type III effectors. In the ongoing evolutionary arms race between hosts and pathogens, plants have countered effector suppression of PTI by evolving the capacity to detect the effectors, inducing effector triggered immunity or ETI [19]. Characterization of the interaction between individual effectors and specific plant proteins and processes is ongoing in several model plant systems including *Arabidopsis thaliana*, tomato, and *Nicotiana benthamiana*. A variety of primary and secondary effects of type III effectors on plant biological processes have been documented using experimental approaches ranging from pull-down assays confirming specific protein-protein interactions to microarray data showing large scale effector-induced changes in plant gene expression [20,21]. Host interactions for other bacterial features including the various PAMPs and toxins such as coronatine are also being characterized, resulting in a large and complex body of literature that describes the intricacies of host-pathogen interaction at the molecular level [22,23].

### 2.4. Regulatory Features and Transcript Identification

Although the type III effectors are a major focus of *P. syringae* research, other genome scale data streams are simultaneously being generated with significant potential to impact our understanding of the larger picture of *P. syringae* metabolism [24-26]. Most significant among these has been the application of transcriptome and proteome sequencing to evaluate gene expression in *Pto* DC3000. The transcriptome mapping has also revealed locations of small RNAs which are linked to gene regulation in numerous biological systems [24]. To date, transcriptome sequencing has focused primarily on transcript profiles of bacteria grown in an iron-limiting media analogous to plant apoplastic fluid. As additional conditions are tested, an increasingly nuanced picture of bacterial gene expression is expected to emerge.

## 3. Challenges and Opportunities in the Analysis and Dissemination of *P. syringae* Data Streams

A major challenge presented by this wealth of data is how to best manage the different data streams for the purpose of maximizing accessibility to users, reducing duplication among data producers, and more efficiently identifying emergent properties of the system. Achievement of these goals further requires that data consolidation be conducted according to consistent standards, that it maintain fidelity and transparency to the information source so that users can rapidly access primary information as needed, and that it keep pace with ongoing research.

Online data repositories presently in existence come in a variety of forms, the best known of which are the large-scale, stably funded data clearinghouses at NCBI, EMBL, and DDBJ. Journal-driven mandates that published sequences be deposited at these sites historically have provided a durable link between sequence data and related publications. However, this system has struggled to accommodate the scale of data generated in the post-sequencing era with a major casualty being the link between ongoing experimental characterization and deposited sequence data. Even the ongoing curation of select genome sequences by Refseq [27], created in 2002 to provide comprehensive and consistently annotated sequence records, remains highly general and rarely incorporates species-specific, experimentally-documented findings. As experimental studies rely increasingly on previously deposited sequence data, and published output often fails to reference the source sequence or produce updates to the annotation record, the link between experimental findings and gene annotation becomes even more tenuous.

In response to the limitations inherent to large generic databases, many model organism research communities maintain organism-specific databases where sequence data can be downloaded, new sequence characterizations added, and tools for genome manipulation made available. Examples include the Pseudomonas Genome Database [28] as well as the SOL Genomics Network (SGN) [29] and The Arabidopsis Information Resource (TAIR) [30], devoted to solanaceous plants and *Arabidopsis*, respectively. These sites are typically maintained by a team of personnel and require significant computational resources, thus being dependent on dedicated funding sources. The model adopted by the *Pseudomonas*-Plant Interaction (PPI) genome resources website [1] is less resource intensive, attempting to address data consolidation needs specific to the *P. syringae* research community through targeted curation of features of particular interest, leveraging existing resources when possible, and minimizing infrastructure.

In practice, the PPI website serves to keep users informed of the many categories of data relevant to *P. syringae* as well as the range of tools available for data analysis. For example, the site maintains a running tally of complete and draft *P. syringae* genome sequences on its main page, Additionally it serves as a repository for datasets that are either too extensive for publication or have been previously published as supplemental files. Examples of these can be found under links to “Comparative genomics and genome organization”, “Plant defense gene characterization”, and “Systems biology and gene expression modeling”, the last of which contains files pertaining to characterization of regulatory features. To facilitate visualization of genome sequences and sequence features, critical to understanding overall genome structure, tutorials have also been provided for use of the Artemis genome viewer and Artemis Comparison Tool (ACT) [31].

### 3.1. Type III Effector Sequence Curation on the PPI Website

A category of data receiving particular attention on the PPI website are the *hop* gene sequences themselves, information on which is summarized in the PPI Hop Database. The rapid burst of *hop* gene characterization following release of the *Pto* DC3000 genome sequence made apparent the need for systematic curation of these genes with emphasis on the need to develop community standards regarding the criteria for gene identification and name assignment. The resulting nomenclature system and criteria on which it is based have proven highly durable in the face of ongoing genome sequencing [32]. Newly characterized *hop* sequences can be submitted to the PPI website directly [1], or characterization and nomenclature assignments performed in collaboration with website personnel. More commonly, website personnel generate independent annotations of draft genomes, from which *hop* gene sequences are identified on the basis of BLAST hits and locations of predicted HrpL binding sites. At present, over 650 individual type III effector genes have been sequenced, composing 59 distinct families. Only full-length *hop* genes from draft genomes are incorporated into the type III effector database except in cases where re-sequencing has confirmed the validity of truncations arising from frameshifts or internal stop codons.

### 3.2. Type III Effector Functional Curation on the PPI Website

As described in section 2.3, biological characterization of the impact of type III effectors on host plants has been conducted over many years using various host plants, a variety of experimental methods, and is described in the literature with inconsistent terminology which itself has evolved as our understanding of the nature of the plant interaction has changed. Subsequent to development of the Hop Database, the *Pseudomonas*-Plant Interaction Resource was added to the PPI website with the goal of systematically capturing the varied data streams relevant to ongoing biological characterization of pathogen gene products interacting with the host. A major question regarding the design of this resource concerned how best to translate and condense the functional data into a more accessible format. While its complexity defies easy comprehension, particularly for newcomers in the field, it is too incomplete to merit diagrammatic representation akin to that used by Kyoto Encyclopedia of Genes and Genomes (KEGG) to represent metabolic pathways [33]. It was ultimately decided that Gene Ontology Annotation (GOA), involving assignment of systematic descriptive terminology to the individual gene products and attached to evidence codes, source publications, and host taxonomy, represented the best strategy for capturing these data [34].

**Figure 1:**
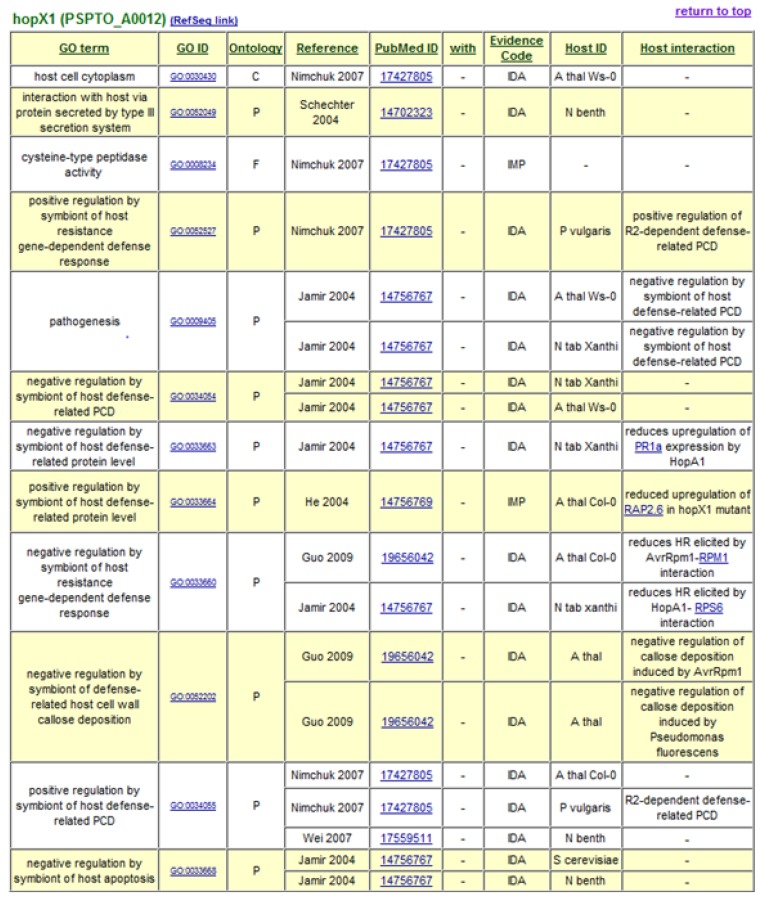
Gene ontology (GO) annotation term assignments for type III effector HopX1 from *P. syringae* pv. *tomato.* GO terms used fall into three separate ontologies corresponding to cellular component (C), molecular function (F), and biological process (P). First author and publication year of the source of the data are shown together with a hyperlinked Pubmed ID. A key to evidence codes can be found at [37].

The decision to use Gene Ontology Annotation was reinforced by the availability of new terms developed by the Plant-Associated Microbe Gene Ontology working group to specifically describe host-symbiont interactions [35]. GO term assignments to *P. syringae* Type III effectors form the basis of the *P. syringae*-Plant Interaction Resource, with terms describing the cellular components or locations to which the effectors are targeted, their molecular functions, and the biological processes in which they engage. A “Host Interaction” field is also included to provide additional descriptive notes and hyperlinks to interacting host proteins. At present, over 600 GO annotation assignments are listed for 50 type III effectors and 8 helper proteins. Gene Ontology annotations generated for type III effector HopX1 are shown in Figure 1. The data can be accessed in a sortable, non-formatted spreadsheet and in formatted tables hyperlinked to interacting proteins, source publications, and the GO database itself. Relevant annotation fields from the *P. syringae*-Plant Interaction Resource are forwarded on to the GO database [36].

### 3.3. PPI Integration with Outside Resources

Consistent with the goal of leveraging existing resources when possible, the PPI site does not host independent annotation records or a dedicated genome viewer but rather functions as a portal for integrating new information on genome features into other, durable supported databases. Data flow typically begins with direct communication of annotation updates from PPI to Genbank, with updates automatically reflected in RefSeq, EMBL and uploaded into the annotation records at the *Pseudomonas* Genome database [28] on a semi-annual basis. At this time, over 3400 annotation changes to *P. syringae* genomes have been forwarded to NCBI via the PPI website. Given that Genbank, Refseq, and EMBL annotation records form the basis for most secondary genome analyses, this approach has the added benefit of maximizing access to the most current research finding, in contrast to many model organism databases where in-house annotation changes are not reliably reflected by the large generic databases.

**Figure 2:**
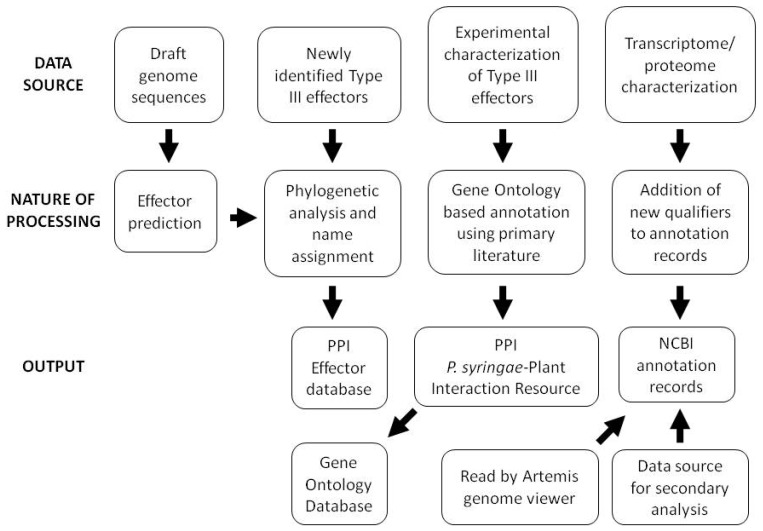
Strategy for data management used at the Pseudomonas-Plant Interaction (PPI) genome resources website [1].

Although corrected *hop* gene nomenclature and mapping of HrpL binding sites were an early focus of annotation updates, proteome and transcriptome sequencing have more recently led to updated gene coordinates, addition of locus qualifiers describing the bioinformatic and experimental support for individual gene calls, and annotation of small RNAs. In light of the constant challenge presented by the large numbers of hypothetical and conserved hypothetical genes in bacterial genomes, incorporation of these qualifiers into the *Pto* DC3000 genome provides invaluable information for those using the *Pto* DC3000 annotation as a basis for annotation of related genomes. An outline of the overall data handling strategy employed for a variety of data types at the PPI website is shown in Figure 2.

### 3.4. PPI Website—Design Philosophy

The PPI website is designed around a simple model for data delivery, depending primarily on flat file formats such as Excel spreadsheets rather than relational databases. While this approach may ultimately shift as the nature of data being produced by the *P. syringae* research community changes, the current strategy has proven easy to maintain with the limited resources and may in fact represent a more flexible model for the volume of data in question. As Matthews et al have pointed out, online resources that permit data access only in response to specific queries can be overly limiting while maintaining a significant degree of browsability permits “the possibility of higher order perusal of data to allow the potential for recognizing unexpected associations” [38]. The durability of this model is further borne out by that fact that the PPI website and component elements have been regularly updated for over nine years, during a period when the average lifetime of biological databases is approximately 18 months [39].

## 4. Conclusions

The larger mission of the PPI website is to orient users to a wide range of *P. syringae* data with particular emphasis on type III effector identification and nomenclature via the Hop Database, and Gene Ontology-based documentation of functional properties as represented in the *P. syringae*-Plant Interaction Resource. The latter is currently being expanded to include annotations for the bacterial features (PAMPs) implicated in triggering innate immunity. A large scale analysis of tomato kinases interacting with PAMPs and individual effectors has been recently initiated and is expected to result in significant expansion of known biological interactions. The ultimate goal of an expanded *P. syringae*-Plant Interaction Resource is the generation of a data network where correlations can be made between repertoires of effectors, functional roles, and host range.

These analyses depend not only on effective data consolidation but also on sufficiently detailed analysis of draft genome sequences. Drafts have the potential to yield important insights on effector repertoires from phenotypically diverse strains; however, re-sequencing of gene fragments to distinguish true effector mutations from assembly errors is critical to the utility of these data.
